# Methyl 6-*O*-trityl-α-d-gluco­pyran­oside methanol disolvate

**DOI:** 10.1107/S1600536814006461

**Published:** 2014-03-29

**Authors:** Zeynep Gültekin, Mehmet Civan, Wolfgang Frey, Tuncer Hökelek

**Affiliations:** a Department of Chemistry, Çankırı Karatekin University, TR-18100, Çankırı, Turkey; b Department of Physics, Hacettepe University, 06800 Beytepe, Ankara, Turkey; c Universität Stuttgart, Pfaffenwaldring 55, D-70569 Stuttgart, Germany

## Abstract

The asymmetric unit of the title compound, C_26_H_28_O_6_·2CH_3_OH, contains two independent methyl 6-*O*-trityl-α-d-gluco­pyran­oside mol­ecules and four methanol solvent mol­ecules. The rings of two methyl α-d-gluco­pyran­oside adopt chair conformations. In the crystal, extensive intra- and inter­molecular O—H⋯O and C—H⋯O hydrogen bonds link the mol­ecules into a three-dimensional supra­molecular architecture.

## Related literature   

For intra­molecular 1,3-dipolar cyclo­additions of various carbohydrates, see: Kobayashi *et al.* (1994 ▶); Kleban *et al.* (2001 ▶); Dransfield *et al.* (1999 ▶); Gallos *et al.* (1999 ▶). For the importance of the title compound for the preparation of di- and tris­accharide analogues after several steps, see: Peri *et al.* (2002 ▶, 2004 ▶); Lopez *et al.* (2011 ▶). For the use of alkyl-5-enyl aldehydes as inter­mediates for the syntheses of bi­cyclo­[*x*.3.0] or bi­cyclo­[*x*.2.1] derivatives after 1,3-dipolar cyclo­additions, see: Dransfield *et al.* (1999 ▶). For the preparation of the title compound, see: Horton & Lauterback (1969 ▶); Bernet & Vasella (1979 ▶); Komiotis *et al.* (2006 ▶). For ring puckering parameters, see: Cremer & Pople (1975 ▶).
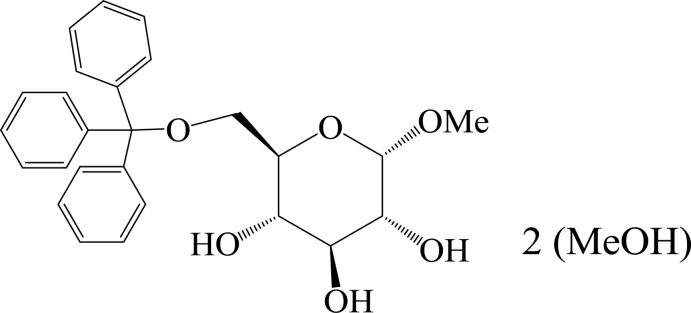


## Experimental   

### 

#### Crystal data  

C_26_H_28_O_6_·2CH_4_O
*M* *_r_* = 500.57
Triclinic, 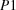

*a* = 8.6763 (4) Å
*b* = 9.1233 (4) Å
*c* = 19.1545 (6) Å
α = 86.328 (3)°
β = 82.578 (2)°
γ = 67.903 (2)°
*V* = 1392.87 (10) Å^3^
*Z* = 2
Mo *K*α radiation
μ = 0.09 mm^−1^
*T* = 299 K
0.44 × 0.41 × 0.22 mm

#### Data collection  

Bruker Kappa APEXII DUO diffractometer
Absorption correction: multi-scan (Blessing, 1995 ▶) *T*_min_ = 0.720, *T*_max_ = 0.745
40377 measured reflections
11390 independent reflections
10449 reflections with *I* > 2σ(*I*)
*R*_int_ = 0.017

#### Refinement  

*R*[*F* ^2^ > 2σ(*F* ^2^)] = 0.035
*wR*(*F* ^2^) = 0.096
*S* = 1.07
11390 reflections
695 parameters
8 restraints
H atoms treated by a mixture of independent and constrained refinement
Δρ_max_ = 0.22 e Å^−3^
Δρ_min_ = −0.24 e Å^−3^

### 

Data collection: *APEX2* (Bruker, 2008 ▶); cell refinement: *SAINT* (Bruker, 2008 ▶); data reduction: *SAINT*; program(s) used to solve structure: *SHELXS97* (Sheldrick, 2008 ▶); program(s) used to refine structure: *SHELXL97* (Sheldrick, 2008 ▶); molecular graphics: *ORTEP-3 for Windows* (Farrugia, 2012 ▶); software used to prepare material for publication: *WinGX* (Farrugia, 2012 ▶) and *PLATON* (Spek, 2009 ▶).

## Supplementary Material

Crystal structure: contains datablock(s) I, global. DOI: 10.1107/S1600536814006461/xu5777sup1.cif

Structure factors: contains datablock(s) I. DOI: 10.1107/S1600536814006461/xu5777Isup2.hkl

CCDC reference: 993298

Additional supporting information:  crystallographic information; 3D view; checkCIF report

## Figures and Tables

**Table 1:** Hydrogen-bond geometry (Å, °)

*D*—H⋯*A*	*D*—H	H⋯*A*	*D*⋯*A*	*D*—H⋯*A*
O3*A*—H31⋯O10	0.84 (3)	1.88 (3)	2.707 (2)	169 (2)
O3*B*—H32⋯O7	0.82 (3)	1.91 (3)	2.713 (2)	165 (3)
O4*A*—H41⋯O2*B*	0.83 (3)	2.02 (2)	2.8287 (18)	164.5 (19)
O4*B*—H42⋯O2*A* ^i^	0.77 (3)	2.15 (3)	2.9046 (19)	166 (3)
O5*A*—H51⋯O8	0.79 (3)	1.87 (3)	2.667 (2)	175 (4)
O5*B*—H52⋯O9^i^	0.81 (4)	1.90 (4)	2.704 (2)	173 (3)
O7—H71⋯O5*A* ^i^	0.79 (2)	1.98 (2)	2.763 (2)	170 (3)
O8—H81⋯O3*B*	0.90 (3)	1.84 (3)	2.727 (2)	170 (3)
O9—H91⋯O3*A*	0.85 (4)	1.91 (4)	2.741 (2)	167 (4)
O10—H101⋯O5*B*	0.81 (3)	1.95 (3)	2.754 (2)	170 (3)
C1*B*—H1*B*⋯O3*A* ^ii^	0.98	2.32	3.2469 (19)	157
C3*A*—H3*A*⋯O4*B* ^iii^	0.98	2.59	3.471 (2)	149
C3*B*—H3*B*⋯O4*A*	0.98	2.49	3.391 (2)	153
C15*B*—H15*B*⋯O1*B*	0.93	2.52	3.417 (2)	161
C25*A*—H25*A*⋯O1*A*	0.93	2.60	3.443 (2)	151

Symmetry codes: (i) 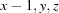; (ii) 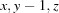; (iii) 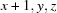.

**Fig. 1.**
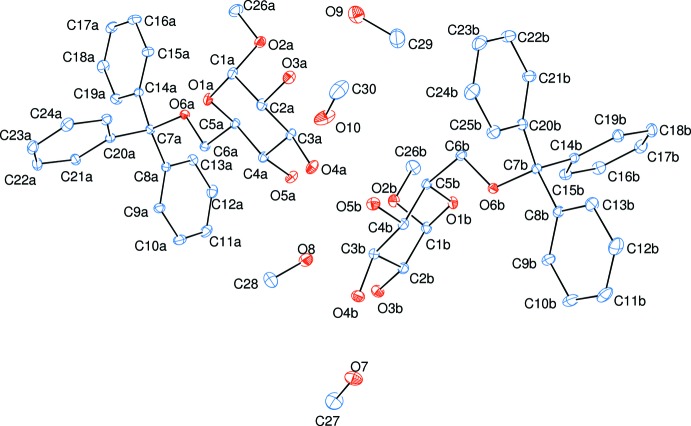
The molecular structure of the title compound with the atom-numbering scheme. Displacement ellipsoids are drawn at the 50% probability level. Hydrogen atoms have been omitted for clarity.

**Fig. 2.**
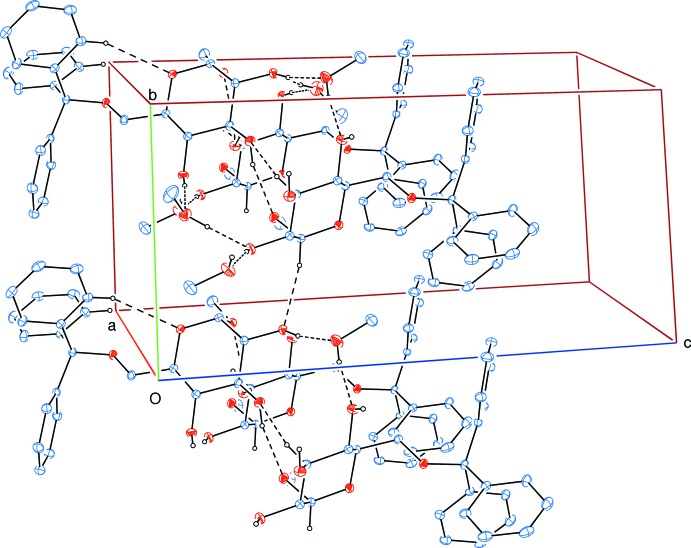
A partial packing diagram. Hydrogen bonds are shown as dashed lines. Hydrogen atoms not involved in hydrogen bonding have been omitted for clarity.

## supplementary crystallographic information

### 1. Comment

The intramolecular 1,3-dipolar cycloadditions of various carbohydrates have been reported by several authors (Kobayashi *et al.*, 1994; Kleban *et al.*, 2001; Dransfield *et al.*, 1999; Gallos *et al.*, 1999). 1,3-Dipolar cycloadducts of various carbohdrates were converted into the amino (hydroxymethyl) cyclopentanetriols which are useful for glycosidase inhibitors (Kleban *et al.*, 2001). The title compound is useful for the preparation of di- and trisaccharide analogues after several steps (Peri *et al.*, 2002, 2004; Lopez *et al.*, 2011), and it can be easily converted to the alkyl-5-enyl aldehydes. These aldehydes are useful intermediates for the syntheses of bicyclo[x.3.0] or bicyclo[x.2.1] derivatives after 1,3-dipolar cycloadditions (Dransfield *et al.*, 1999). The present study was undertaken to ascertain the crystal structure of the title compound.

The asymmetric unit of the title compound contains two crystallographically independent molecules and four methanol solvent molecules (Fig. 1). The methyl α-D-glucopyranoside rings [*A* (O1a/C1a–C5a) and *A'* (O1b/C1b–C5b)] are in chair conformations with puckering parameters (Cremer & Pople, 1975) of Q_T_ = 0.551 (2)Å, φ = -5.8(2.5)°, θ = 4.4 (2)° and Q_T_ = 0.550 (2)Å, φ = -40.7(4.9)°, θ = 2.0 (2)°, respectively. In trityl cation, the rings *B* (C8a–C13a), *C* (C14a–C19a), *D* (C20a–C25a) and *B'* (C8b–C13b), *C'* (C14b–C19b), *D'*(C20b–C25b) are oriented at dihedral angles of A/B = 74.16 (6), A/C = 63.29 (7), B/C = 83.19 (6) ° and A'/B' = 82.73 (6), A'/C' = 73.69 (7), B'/C' = 65.34 (6) °, respectively. The intramolecular O—H···O and C—H···O hydrogen bonds (Table 1) link the molecules in the asymmetric unit.

In the crystal structure, intra- and intermolecular O-H···O and C-H···O hydrogen bonds (Table 1, Fig. 2) link the molecules into a three-dimensional structure.

### 2. Experimental

The title compound was synthesized by the literature method (Horton *et al.*, 1969; Bernet & Vasella, 1979; Komiotis *et al.*, 2006). α-D-glucopyranoside (10.0 g, 51.49 mmol) and triphenyl methyl chloride (14.4 g, 52.0 mmol) in pyridine (100 ml) was heated for 3 h at 373 K under argon atmosphere, and then the yellowish solution was stirred overnight at room temperature for 24 h. Pyridine was removed in vacuo and water (100 ml) was added to the mixture. The crude mixture was extracted with EtOAc (4 × 50 ml). Combined organic layer was dried over MgSO_4_, filtered and the solvent was removed in vacuo. Crystallization of the crude product in MeOH:petroleum ether (5:1) gave a colorless crystalline solid (yield: 80%), m.p.: 410–411 K, literature m.p.: 421-422 K (Bernet & Vasella, 1979).

### Crystal data

**Table:**

C_26_H_28_O_6_·2CH_4_O	*Z* = 2
*M**_r_* = 500.57	*F*(000) = 536
Triclinic, *P*1	*D*_x_ = 1.194 Mg m^−^^3^
Hall symbol: P 1	Mo *K*α radiation, λ = 0.71073 Å
*a* = 8.6763 (4) Å	Cell parameters from 10444 reflections
*b* = 9.1233 (4) Å	θ = 2.1–26.5°
*c* = 19.1545 (6) Å	µ = 0.09 mm^−^^1^
α = 86.328 (3)°	*T* = 299 K
β = 82.578 (2)°	Block, colourless
γ = 67.903 (2)°	0.44 × 0.41 × 0.22 mm
*V* = 1392.87 (10) Å^3^	

### Data collection

**Table:**

Bruker Kappa APEXII DUO diffractometer	11390 independent reflections
Radiation source: fine-focus sealed tube	10449 reflections with *I* > 2σ(*I*)
Triumph monochromator	*R*_int_ = 0.017
ω + Phi Scans scans	θ_max_ = 26.5°, θ_min_ = 2.1°
Absorption correction: multi-scan (Blessing, 1995)	*h* = −10→10
*T*_min_ = 0.720, *T*_max_ = 0.745	*k* = −11→11
40377 measured reflections	*l* = −23→23

### Refinement

**Table:**

Refinement on *F*^2^	Primary atom site location: structure-invariant direct methods
Least-squares matrix: full	Secondary atom site location: difference Fourier map
*R*[*F*^2^ > 2σ(*F*^2^)] = 0.035	Hydrogen site location: inferred from neighbouring sites
*wR*(*F*^2^) = 0.096	H atoms treated by a mixture of independent and constrained refinement
*S* = 1.07	*w* = 1/[σ^2^(*F*_o_^2^) + (0.0559*P*)^2^ + 0.1152*P*] where *P* = (*F*_o_^2^ + 2*F*_c_^2^)/3
11390 reflections	(Δ/σ)_max_ = 0.001
695 parameters	Δρ_max_ = 0.22 e Å^−^^3^
8 restraints	Δρ_min_ = −0.24 e Å^−^^3^

### Special details

**Table:**

Geometry. All esds (except the esd in the dihedral angle between two l.s. planes) are estimated using the full covariance matrix. The cell esds are taken into account individually in the estimation of esds in distances, angles and torsion angles; correlations between esds in cell parameters are only used when they are defined by crystal symmetry. An approximate (isotropic) treatment of cell esds is used for estimating esds involving l.s. planes.
Refinement. Refinement of F^2^ against ALL reflections. The weighted R-factor wR and goodness of fit S are based on F^2^, conventional R-factors R are based on F, with F set to zero for negative F^2^. The threshold expression of F^2^ > 2sigma(F^2^) is used only for calculating R-factors(gt) etc. and is not relevant to the choice of reflections for refinement. R-factors based on F^2^ are statistically about twice as large as those based on F, and R- factors based on ALL data will be even larger.

### Fractional atomic coordinates and isotropic or equivalent isotropic displacement parameters (Å^2^)

**Table:**

	*x*	*y*	*z*	*U*_iso_*/*U*_eq_	
O1A	0.67336 (14)	0.99787 (12)	0.09862 (5)	0.0469 (3)	
O2A	0.77130 (15)	0.98268 (14)	0.20819 (6)	0.0500 (3)	
O3A	0.46073 (17)	1.02657 (14)	0.27635 (6)	0.0524 (3)	
H31	0.369 (3)	1.024 (3)	0.2948 (12)	0.063 (6)*	
O4A	0.40182 (15)	0.77468 (16)	0.21955 (7)	0.0558 (3)	
H41	0.428 (2)	0.678 (3)	0.2258 (10)	0.053 (5)*	
O5A	0.67937 (16)	0.59516 (13)	0.11406 (7)	0.0540 (3)	
H51	0.603 (3)	0.570 (4)	0.1095 (17)	0.109 (11)*	
O6A	0.92014 (12)	0.84787 (12)	−0.01329 (5)	0.0395 (2)	
C1A	0.6321 (2)	1.04331 (18)	0.16985 (8)	0.0455 (4)	
H1A	0.5887	1.1591	0.1715	0.055*	
C2A	0.4962 (2)	0.98605 (18)	0.20406 (8)	0.0435 (3)	
H2A	0.3946	1.0410	0.1811	0.052*	
C3A	0.54653 (19)	0.81009 (18)	0.19520 (8)	0.0426 (3)	
H3A	0.6359	0.7528	0.2245	0.051*	
C4A	0.60696 (19)	0.76254 (17)	0.11881 (8)	0.0417 (3)	
H4A	0.5117	0.8032	0.0911	0.050*	
C5A	0.7386 (2)	0.82993 (17)	0.08876 (8)	0.0403 (3)	
H5A	0.8398	0.7802	0.1125	0.048*	
C6A	0.7810 (2)	0.80281 (19)	0.01065 (8)	0.0420 (3)	
H61	0.8092	0.6921	0.0009	0.050*	
H62	0.6854	0.8654	−0.0137	0.050*	
C7A	0.95558 (17)	0.85998 (17)	−0.08845 (7)	0.0361 (3)	
C8A	0.97374 (18)	0.70260 (18)	−0.11965 (7)	0.0391 (3)	
C9A	0.8427 (2)	0.6814 (2)	−0.14656 (9)	0.0507 (4)	
H9A	0.7465	0.7687	−0.1536	0.061*	
C10A	0.8531 (3)	0.5325 (3)	−0.16304 (10)	0.0630 (5)	
H10A	0.7639	0.5206	−0.1810	0.076*	
C11A	0.9938 (3)	0.4019 (2)	−0.15317 (11)	0.0669 (5)	
H11A	0.9999	0.3017	−0.1638	0.080*	
C12A	1.1259 (3)	0.4207 (2)	−0.12731 (11)	0.0631 (5)	
H12A	1.2222	0.3330	−0.1209	0.076*	
C13A	1.1161 (2)	0.5697 (2)	−0.11083 (9)	0.0491 (4)	
H13A	1.2064	0.5809	−0.0936	0.059*	
C14A	1.11908 (17)	0.89096 (17)	−0.10031 (8)	0.0391 (3)	
C15A	1.1670 (2)	0.9576 (2)	−0.04791 (10)	0.0500 (4)	
H15A	1.1047	0.9754	−0.0038	0.060*	
C16A	1.3061 (2)	0.9976 (2)	−0.06079 (12)	0.0609 (5)	
H16A	1.3378	1.0407	−0.0250	0.073*	
C17A	1.3989 (2)	0.9746 (2)	−0.12582 (12)	0.0594 (5)	
H17A	1.4915	1.0039	−0.1344	0.071*	
C18A	1.3531 (2)	0.9083 (3)	−0.17757 (11)	0.0649 (5)	
H18A	1.4155	0.8917	−0.2216	0.078*	
C19A	1.2146 (2)	0.8655 (2)	−0.16514 (9)	0.0559 (4)	
H19A	1.1857	0.8193	−0.2007	0.067*	
C20A	0.82246 (17)	1.00806 (18)	−0.11745 (8)	0.0400 (3)	
C21A	0.8104 (2)	1.0315 (2)	−0.18955 (9)	0.0488 (4)	
H21A	0.8777	0.9529	−0.2209	0.059*	
C22A	0.6979 (2)	1.1721 (2)	−0.21449 (11)	0.0607 (5)	
H22A	0.6912	1.1873	−0.2626	0.073*	
C23A	0.5971 (2)	1.2884 (2)	−0.16949 (13)	0.0652 (5)	
H23A	0.5202	1.3809	−0.1868	0.078*	
C24A	0.6096 (2)	1.2681 (2)	−0.09873 (13)	0.0634 (5)	
H24A	0.5418	1.3480	−0.0681	0.076*	
C25A	0.7224 (2)	1.12958 (19)	−0.07223 (10)	0.0493 (4)	
H25A	0.7311	1.1180	−0.0241	0.059*	
C26A	0.9017 (3)	1.0390 (3)	0.18293 (12)	0.0714 (5)	
H261	0.9538	0.9937	0.1380	0.107*	
H263	0.9836	1.0090	0.2157	0.107*	
H262	0.8552	1.1522	0.1782	0.107*	
O1B	0.31293 (14)	0.45350 (12)	0.37621 (5)	0.0440 (2)	
O2B	0.47236 (14)	0.45945 (14)	0.26845 (6)	0.0500 (3)	
O3B	0.25027 (18)	0.39417 (14)	0.19786 (6)	0.0551 (3)	
H32	0.163 (3)	0.406 (3)	0.1816 (12)	0.062 (6)*	
O4B	−0.05021 (16)	0.65235 (17)	0.24352 (7)	0.0553 (3)	
H42	−0.090 (3)	0.743 (3)	0.2394 (12)	0.069 (7)*	
O5B	−0.00591 (17)	0.84545 (13)	0.35104 (7)	0.0526 (3)	
H52	−0.107 (4)	0.874 (3)	0.3576 (14)	0.084 (8)*	
O6B	0.07427 (12)	0.59786 (12)	0.49097 (5)	0.0394 (2)	
C1B	0.3571 (2)	0.39980 (18)	0.30618 (8)	0.0431 (3)	
H1B	0.4087	0.2840	0.3073	0.052*	
C2B	0.2021 (2)	0.44796 (18)	0.26809 (8)	0.0445 (3)	
H2B	0.1308	0.3936	0.2915	0.053*	
C3B	0.10178 (19)	0.62449 (17)	0.27215 (8)	0.0416 (3)	
H3B	0.1643	0.6819	0.2440	0.050*	
C4B	0.06732 (18)	0.67721 (16)	0.34836 (8)	0.0407 (3)	
H4B	−0.0100	0.6326	0.3746	0.049*	
C5B	0.22883 (19)	0.62183 (17)	0.38259 (8)	0.0385 (3)	
H5B	0.3019	0.6741	0.3589	0.046*	
C6B	0.19910 (19)	0.65638 (18)	0.46040 (8)	0.0402 (3)	
H63	0.3018	0.6044	0.4819	0.048*	
H64	0.1613	0.7694	0.4676	0.048*	
C7B	0.04814 (17)	0.58957 (16)	0.56649 (7)	0.0356 (3)	
C8B	−0.11496 (17)	0.55913 (17)	0.58210 (8)	0.0394 (3)	
C9B	−0.1615 (2)	0.4821 (2)	0.53349 (10)	0.0507 (4)	
H9B	−0.0971	0.4555	0.4901	0.061*	
C10B	−0.3025 (2)	0.4443 (2)	0.54856 (13)	0.0646 (5)	
H10B	−0.3328	0.3941	0.5150	0.078*	
C11B	−0.3973 (2)	0.4798 (2)	0.61202 (14)	0.0682 (6)	
H11B	−0.4910	0.4525	0.6221	0.082*	
C12B	−0.3539 (2)	0.5559 (3)	0.66101 (13)	0.0708 (6)	
H12B	−0.4184	0.5805	0.7044	0.085*	
C13B	−0.2129 (2)	0.5970 (2)	0.64601 (10)	0.0564 (4)	
H13B	−0.1851	0.6500	0.6793	0.068*	
C14B	0.18515 (17)	0.44195 (17)	0.59409 (8)	0.0371 (3)	
C15B	0.2934 (2)	0.32726 (18)	0.54781 (9)	0.0462 (3)	
H15B	0.2885	0.3434	0.4996	0.055*	
C16B	0.4089 (2)	0.1890 (2)	0.57281 (11)	0.0576 (4)	
H16B	0.4813	0.1138	0.5411	0.069*	
C17B	0.4176 (2)	0.1617 (2)	0.64408 (11)	0.0602 (5)	
H17B	0.4956	0.0688	0.6605	0.072*	
C18B	0.3093 (2)	0.2737 (2)	0.69066 (10)	0.0572 (4)	
H18B	0.3133	0.2558	0.7389	0.069*	
C19B	0.1945 (2)	0.4127 (2)	0.66593 (9)	0.0468 (4)	
H19B	0.1226	0.4876	0.6979	0.056*	
C20B	0.03483 (18)	0.74705 (18)	0.59505 (8)	0.0382 (3)	
C21B	0.1630 (2)	0.7665 (2)	0.62465 (10)	0.0520 (4)	
H21B	0.2559	0.6783	0.6345	0.062*	
C22B	0.1543 (3)	0.9163 (3)	0.63984 (11)	0.0672 (5)	
H22B	0.2419	0.9274	0.6595	0.081*	
C23B	0.0185 (3)	1.0479 (2)	0.62629 (11)	0.0683 (6)	
H23B	0.0134	1.1480	0.6365	0.082*	
C24B	−0.1096 (3)	1.0302 (2)	0.59751 (12)	0.0690 (5)	
H24B	−0.2025	1.1190	0.5882	0.083*	
C25B	−0.1021 (2)	0.8817 (2)	0.58215 (10)	0.0534 (4)	
H25B	−0.1906	0.8718	0.5628	0.064*	
C26B	0.6300 (2)	0.4008 (3)	0.29548 (12)	0.0728 (6)	
H264	0.6175	0.4420	0.3416	0.109*	
H265	0.7083	0.4336	0.2649	0.109*	
H266	0.6706	0.2874	0.2982	0.109*	
O7	−0.0018 (2)	0.3825 (2)	0.13088 (9)	0.0811 (4)	
H71	−0.092 (2)	0.449 (3)	0.1299 (13)	0.082 (9)*	
O8	0.4376 (2)	0.4936 (2)	0.09441 (9)	0.0781 (4)	
O9	0.6599 (2)	0.9282 (2)	0.38342 (12)	0.0916 (5)	
O10	0.1595 (2)	1.0518 (2)	0.34549 (10)	0.0950 (6)	
C27	0.0464 (4)	0.3127 (4)	0.06554 (15)	0.1008 (9)	
H271	0.0039	0.3911	0.0297	0.151*	
H272	0.0023	0.2310	0.0642	0.151*	
H273	0.1664	0.2676	0.0573	0.151*	
C28	0.3102 (5)	0.6022 (5)	0.05810 (18)	0.1225 (12)	
H281	0.2410	0.6870	0.0887	0.184*	
H282	0.2433	0.5497	0.0429	0.184*	
H283	0.3594	0.6440	0.0178	0.184*	
H81	0.387 (3)	0.455 (3)	0.1307 (15)	0.087 (8)*	
C29	0.5938 (4)	0.8244 (5)	0.42249 (18)	0.1127 (10)	
H291	0.6489	0.7889	0.4642	0.169*	
H292	0.6115	0.7348	0.3943	0.169*	
H293	0.4759	0.8787	0.4355	0.169*	
H91	0.613 (5)	0.953 (5)	0.346 (2)	0.130 (14)*	
C30	0.0938 (5)	1.1398 (3)	0.40562 (15)	0.1068 (10)	
H301	0.1386	1.2213	0.4054	0.160*	
H302	0.1228	1.0722	0.4463	0.160*	
H303	−0.0259	1.1871	0.4071	0.160*	
H101	0.120 (4)	0.985 (3)	0.3435 (16)	0.103 (10)*	

### Atomic displacement parameters (Å^2^)

**Table:**

	*U*^11^	*U*^22^	*U*^33^	*U*^12^	*U*^13^	*U*^23^
O1A	0.0607 (7)	0.0393 (5)	0.0398 (6)	−0.0214 (5)	0.0035 (5)	0.0041 (4)
O2A	0.0580 (7)	0.0526 (6)	0.0460 (6)	−0.0301 (5)	0.0008 (5)	−0.0012 (5)
O3A	0.0587 (7)	0.0506 (6)	0.0464 (6)	−0.0227 (5)	0.0109 (5)	−0.0090 (5)
O4A	0.0516 (7)	0.0501 (7)	0.0653 (7)	−0.0254 (5)	0.0160 (5)	−0.0013 (5)
O5A	0.0555 (7)	0.0428 (6)	0.0648 (7)	−0.0238 (5)	0.0098 (5)	−0.0077 (5)
O6A	0.0387 (5)	0.0512 (6)	0.0327 (5)	−0.0224 (5)	−0.0017 (4)	0.0002 (4)
C1A	0.0581 (10)	0.0359 (7)	0.0424 (8)	−0.0202 (7)	0.0025 (7)	0.0004 (6)
C2A	0.0467 (8)	0.0408 (8)	0.0393 (8)	−0.0148 (6)	0.0040 (6)	−0.0018 (6)
C3A	0.0416 (7)	0.0436 (7)	0.0428 (7)	−0.0196 (6)	0.0047 (6)	0.0021 (6)
C4A	0.0448 (8)	0.0399 (7)	0.0410 (7)	−0.0180 (6)	0.0011 (6)	−0.0014 (5)
C5A	0.0440 (8)	0.0403 (7)	0.0373 (7)	−0.0179 (6)	−0.0008 (6)	0.0006 (6)
C6A	0.0440 (8)	0.0526 (8)	0.0364 (7)	−0.0278 (7)	0.0015 (6)	−0.0008 (6)
C7A	0.0330 (7)	0.0448 (7)	0.0321 (7)	−0.0169 (6)	−0.0014 (5)	0.0006 (5)
C8A	0.0405 (8)	0.0474 (8)	0.0328 (7)	−0.0216 (6)	0.0003 (6)	−0.0001 (6)
C9A	0.0488 (9)	0.0592 (10)	0.0519 (9)	−0.0290 (8)	−0.0051 (7)	−0.0018 (7)
C10A	0.0741 (13)	0.0713 (12)	0.0601 (11)	−0.0455 (11)	−0.0033 (9)	−0.0106 (9)
C11A	0.0933 (16)	0.0548 (11)	0.0603 (11)	−0.0403 (11)	0.0093 (11)	−0.0116 (8)
C12A	0.0747 (13)	0.0458 (9)	0.0598 (11)	−0.0152 (9)	0.0024 (9)	−0.0028 (8)
C13A	0.0465 (9)	0.0498 (9)	0.0478 (8)	−0.0146 (7)	−0.0044 (7)	−0.0017 (7)
C14A	0.0329 (7)	0.0418 (7)	0.0446 (8)	−0.0163 (6)	−0.0065 (6)	0.0045 (6)
C15A	0.0442 (8)	0.0528 (9)	0.0561 (9)	−0.0218 (7)	−0.0012 (7)	−0.0094 (7)
C16A	0.0461 (9)	0.0599 (10)	0.0847 (13)	−0.0258 (8)	−0.0111 (9)	−0.0125 (9)
C17A	0.0350 (8)	0.0511 (9)	0.0938 (14)	−0.0194 (7)	−0.0047 (9)	0.0038 (9)
C18A	0.0463 (10)	0.0843 (13)	0.0650 (12)	−0.0311 (9)	0.0080 (8)	0.0069 (10)
C19A	0.0453 (9)	0.0831 (13)	0.0472 (9)	−0.0348 (9)	0.0003 (7)	0.0004 (8)
C20A	0.0323 (7)	0.0464 (8)	0.0460 (8)	−0.0211 (6)	−0.0037 (6)	0.0046 (6)
C21A	0.0418 (8)	0.0609 (10)	0.0457 (9)	−0.0224 (7)	−0.0067 (7)	0.0092 (7)
C22A	0.0528 (10)	0.0721 (12)	0.0614 (11)	−0.0291 (9)	−0.0167 (9)	0.0268 (9)
C23A	0.0461 (9)	0.0555 (10)	0.0935 (15)	−0.0190 (8)	−0.0185 (9)	0.0258 (10)
C24A	0.0494 (10)	0.0484 (9)	0.0873 (15)	−0.0144 (8)	−0.0026 (9)	0.0013 (9)
C25A	0.0462 (9)	0.0483 (9)	0.0551 (10)	−0.0197 (7)	−0.0051 (7)	−0.0001 (7)
C26A	0.0729 (13)	0.0846 (14)	0.0736 (12)	−0.0527 (12)	0.0053 (10)	−0.0023 (10)
O1B	0.0516 (6)	0.0404 (5)	0.0357 (5)	−0.0132 (5)	−0.0033 (4)	0.0024 (4)
O2B	0.0462 (6)	0.0517 (6)	0.0476 (6)	−0.0155 (5)	0.0011 (5)	0.0006 (5)
O3B	0.0622 (8)	0.0591 (7)	0.0436 (6)	−0.0208 (6)	−0.0053 (5)	−0.0110 (5)
O4B	0.0563 (7)	0.0523 (7)	0.0610 (7)	−0.0197 (6)	−0.0206 (6)	−0.0011 (5)
O5B	0.0505 (7)	0.0426 (6)	0.0617 (7)	−0.0131 (5)	−0.0065 (5)	−0.0060 (5)
O6B	0.0424 (5)	0.0494 (6)	0.0332 (5)	−0.0254 (5)	−0.0034 (4)	0.0002 (4)
C1B	0.0503 (9)	0.0349 (7)	0.0402 (8)	−0.0129 (6)	−0.0009 (6)	−0.0006 (6)
C2B	0.0532 (9)	0.0431 (8)	0.0395 (8)	−0.0214 (7)	−0.0019 (6)	−0.0034 (6)
C3B	0.0457 (8)	0.0427 (7)	0.0398 (7)	−0.0200 (6)	−0.0078 (6)	0.0025 (6)
C4B	0.0429 (8)	0.0390 (7)	0.0418 (7)	−0.0182 (6)	−0.0015 (6)	−0.0003 (5)
C5B	0.0422 (7)	0.0402 (7)	0.0360 (7)	−0.0206 (6)	0.0020 (6)	−0.0009 (5)
C6B	0.0409 (8)	0.0462 (8)	0.0395 (8)	−0.0239 (6)	−0.0011 (6)	−0.0016 (6)
C7B	0.0310 (7)	0.0432 (7)	0.0346 (7)	−0.0171 (6)	−0.0021 (5)	0.0023 (6)
C8B	0.0316 (7)	0.0400 (7)	0.0474 (8)	−0.0148 (6)	−0.0069 (6)	0.0079 (6)
C9B	0.0453 (9)	0.0496 (9)	0.0638 (10)	−0.0249 (7)	−0.0074 (7)	0.0000 (7)
C10B	0.0497 (10)	0.0568 (10)	0.0988 (16)	−0.0304 (9)	−0.0179 (10)	0.0036 (10)
C11B	0.0387 (9)	0.0571 (10)	0.1128 (17)	−0.0258 (8)	−0.0073 (10)	0.0174 (11)
C12B	0.0452 (10)	0.0830 (14)	0.0801 (14)	−0.0270 (10)	0.0118 (9)	0.0099 (11)
C13B	0.0452 (9)	0.0735 (12)	0.0534 (10)	−0.0288 (8)	0.0035 (7)	0.0020 (8)
C14B	0.0312 (7)	0.0411 (7)	0.0433 (8)	−0.0186 (6)	−0.0049 (6)	0.0036 (6)
C15B	0.0441 (8)	0.0439 (8)	0.0509 (9)	−0.0161 (7)	−0.0058 (7)	−0.0042 (7)
C16B	0.0469 (9)	0.0463 (9)	0.0747 (12)	−0.0116 (7)	−0.0055 (8)	−0.0058 (8)
C17B	0.0446 (9)	0.0495 (9)	0.0840 (14)	−0.0147 (7)	−0.0170 (9)	0.0174 (9)
C18B	0.0497 (10)	0.0669 (11)	0.0572 (10)	−0.0245 (8)	−0.0158 (8)	0.0214 (8)
C19B	0.0400 (8)	0.0542 (9)	0.0456 (9)	−0.0182 (7)	−0.0033 (6)	0.0054 (7)
C20B	0.0391 (7)	0.0434 (7)	0.0334 (7)	−0.0180 (6)	0.0010 (6)	−0.0021 (5)
C21B	0.0512 (9)	0.0520 (9)	0.0589 (10)	−0.0237 (8)	−0.0130 (8)	−0.0016 (7)
C22B	0.0819 (14)	0.0739 (13)	0.0664 (12)	−0.0504 (12)	−0.0126 (10)	−0.0058 (10)
C23B	0.0938 (16)	0.0496 (10)	0.0679 (12)	−0.0366 (11)	0.0044 (11)	−0.0116 (9)
C24B	0.0683 (12)	0.0430 (10)	0.0872 (15)	−0.0132 (9)	−0.0016 (11)	−0.0034 (9)
C25B	0.0487 (9)	0.0473 (9)	0.0624 (11)	−0.0159 (7)	−0.0073 (8)	0.0004 (7)
C26B	0.0458 (10)	0.0862 (14)	0.0765 (13)	−0.0139 (9)	−0.0052 (9)	−0.0009 (11)
O7	0.0757 (11)	0.0799 (10)	0.0832 (10)	−0.0174 (9)	−0.0141 (8)	−0.0317 (8)
O8	0.0870 (11)	0.0884 (11)	0.0752 (10)	−0.0520 (9)	−0.0081 (8)	0.0026 (8)
O9	0.0637 (10)	0.1045 (13)	0.0961 (13)	−0.0189 (9)	−0.0153 (9)	0.0075 (10)
O10	0.0941 (12)	0.1056 (13)	0.1018 (13)	−0.0662 (11)	0.0450 (10)	−0.0514 (10)
C27	0.125 (2)	0.0942 (19)	0.0813 (16)	−0.0400 (17)	0.0087 (15)	−0.0334 (14)
C28	0.149 (3)	0.160 (3)	0.097 (2)	−0.094 (3)	−0.062 (2)	0.051 (2)
C29	0.108 (2)	0.132 (3)	0.101 (2)	−0.050 (2)	−0.0187 (17)	0.0295 (19)
C30	0.148 (3)	0.0887 (17)	0.0921 (18)	−0.0670 (18)	0.0464 (18)	−0.0358 (14)

### Geometric parameters (Å, º)

**Table:**

O1A—C1A	1.4130 (19)	C1B—H1B	0.9800
O1A—C5A	1.4355 (18)	C2B—O3B	1.4179 (19)
O2A—C26A	1.429 (2)	C2B—C3B	1.517 (2)
O3A—H31	0.83 (2)	C2B—H2B	0.9800
O4A—H41	0.83 (2)	C3B—O4B	1.4216 (19)
O5A—H51	0.795 (18)	C3B—C4B	1.523 (2)
O6A—C6A	1.4296 (17)	C3B—H3B	0.9800
O6A—C7A	1.4389 (17)	C4B—O5B	1.4248 (18)
C1A—O2A	1.407 (2)	C4B—C5B	1.520 (2)
C1A—C2A	1.520 (2)	C4B—H4B	0.9800
C1A—H1A	0.9800	C5B—C6B	1.512 (2)
C2A—O3A	1.4209 (19)	C5B—H5B	0.9800
C2A—C3A	1.512 (2)	C6B—H63	0.9700
C2A—H2A	0.9800	C6B—H64	0.9700
C3A—O4A	1.4258 (18)	C7B—C20B	1.529 (2)
C3A—C4A	1.522 (2)	C7B—C8B	1.5313 (19)
C3A—H3A	0.9800	C7B—C14B	1.540 (2)
C4A—O5A	1.4199 (18)	C8B—C13B	1.380 (2)
C4A—C5A	1.525 (2)	C8B—C9B	1.385 (2)
C4A—H4A	0.9800	C9B—C10B	1.383 (2)
C5A—C6A	1.507 (2)	C9B—H9B	0.9300
C5A—H5A	0.9800	C10B—C11B	1.360 (3)
C6A—H61	0.9700	C10B—H10B	0.9300
C6A—H62	0.9700	C11B—C12B	1.370 (4)
C7A—C14A	1.5342 (19)	C11B—H11B	0.9300
C7A—C8A	1.536 (2)	C12B—C13B	1.400 (3)
C7A—C20A	1.537 (2)	C12B—H12B	0.9300
C8A—C9A	1.388 (2)	C13B—H13B	0.9300
C8A—C13A	1.388 (2)	C14B—C15B	1.388 (2)
C9A—C10A	1.382 (3)	C14B—C19B	1.392 (2)
C9A—H9A	0.9300	C15B—C16B	1.385 (2)
C10A—C11A	1.372 (3)	C15B—H15B	0.9300
C10A—H10A	0.9300	C16B—C17B	1.379 (3)
C11A—C12A	1.376 (3)	C16B—H16B	0.9300
C11A—H11A	0.9300	C17B—C18B	1.379 (3)
C12A—C13A	1.384 (3)	C17B—H17B	0.9300
C12A—H12A	0.9300	C18B—C19B	1.385 (2)
C13A—H13A	0.9300	C18B—H18B	0.9300
C14A—C19A	1.382 (2)	C19B—H19B	0.9300
C14A—C15A	1.388 (2)	C20B—C21B	1.384 (2)
C15A—C16A	1.376 (2)	C20B—C25B	1.386 (2)
C15A—H15A	0.9300	C21B—C22B	1.388 (3)
C16A—C17A	1.375 (3)	C21B—H21B	0.9300
C16A—H16A	0.9300	C22B—C23B	1.368 (3)
C17A—C18A	1.366 (3)	C22B—H22B	0.9300
C17A—H17A	0.9300	C23B—C24B	1.367 (3)
C18A—C19A	1.386 (3)	C23B—H23B	0.9300
C18A—H18A	0.9300	C24B—C25B	1.381 (3)
C19A—H19A	0.9300	C24B—H24B	0.9300
C20A—C25A	1.392 (2)	C25B—H25B	0.9300
C20A—C21A	1.395 (2)	C26B—H264	0.9600
C21A—C22A	1.389 (3)	C26B—H265	0.9600
C21A—H21A	0.9300	C26B—H266	0.9600
C22A—C23A	1.363 (3)	O7—C27	1.383 (3)
C22A—H22A	0.9300	O7—H71	0.789 (16)
C23A—C24A	1.369 (3)	O8—C28	1.404 (4)
C23A—H23A	0.9300	O8—H81	0.90 (3)
C24A—C25A	1.389 (3)	O9—C29	1.412 (4)
C24A—H24A	0.9300	O9—H91	0.85 (4)
C25A—H25A	0.9300	O10—C30	1.379 (3)
C26A—H261	0.9600	O10—H101	0.802 (18)
C26A—H263	0.9600	C27—H271	0.9600
C26A—H262	0.9600	C27—H272	0.9600
O1B—C1B	1.4130 (18)	C27—H273	0.9600
O1B—C5B	1.4365 (18)	C28—H281	0.9600
O2B—C26B	1.422 (2)	C28—H282	0.9600
O3B—H32	0.82 (2)	C28—H283	0.9600
O4B—H42	0.77 (3)	C29—H291	0.9600
O5B—H52	0.81 (3)	C29—H292	0.9600
O6B—C6B	1.4254 (17)	C29—H293	0.9600
O6B—C7B	1.4366 (17)	C30—H301	0.9600
C1B—O2B	1.411 (2)	C30—H302	0.9600
C1B—C2B	1.517 (2)	C30—H303	0.9600
			
C1A—O1A—C5A	114.19 (11)	O3B—C2B—H2B	107.7
C1A—O2A—C26A	113.39 (14)	C1B—C2B—H2B	107.7
C2A—O3A—H31	113.4 (15)	C3B—C2B—C1B	111.89 (12)
C3A—O4A—H41	110.3 (14)	C3B—C2B—H2B	107.7
C4A—O5A—H51	104 (2)	O4B—C3B—C2B	106.85 (12)
C6A—O6A—C7A	115.66 (10)	O4B—C3B—C4B	110.88 (12)
O1A—C1A—C2A	109.59 (13)	O4B—C3B—H3B	109.6
O1A—C1A—H1A	108.6	C2B—C3B—C4B	110.14 (12)
O2A—C1A—O1A	112.26 (13)	C2B—C3B—H3B	109.6
O2A—C1A—C2A	109.20 (12)	C4B—C3B—H3B	109.6
O2A—C1A—H1A	108.6	O5B—C4B—C3B	109.90 (12)
C2A—C1A—H1A	108.6	O5B—C4B—C5B	108.42 (12)
O3A—C2A—C1A	109.07 (13)	O5B—C4B—H4B	109.3
O3A—C2A—C3A	111.23 (12)	C3B—C4B—H4B	109.3
O3A—C2A—H2A	108.3	C5B—C4B—C3B	110.66 (12)
C3A—C2A—C1A	111.44 (12)	C5B—C4B—H4B	109.3
C3A—C2A—H2A	108.3	O1B—C5B—C4B	110.07 (11)
C1A—C2A—H2A	108.3	O1B—C5B—C6B	106.67 (12)
O4A—C3A—C2A	106.33 (12)	O1B—C5B—H5B	109.1
O4A—C3A—C4A	110.87 (12)	C4B—C5B—H5B	109.1
O4A—C3A—H3A	109.3	C6B—C5B—C4B	112.63 (12)
C2A—C3A—C4A	111.59 (12)	C6B—C5B—H5B	109.1
C2A—C3A—H3A	109.3	O6B—C6B—C5B	107.27 (11)
C4A—C3A—H3A	109.3	O6B—C6B—H63	110.3
O5A—C4A—C3A	110.08 (12)	O6B—C6B—H64	110.3
O5A—C4A—C5A	108.50 (12)	C5B—C6B—H63	110.3
O5A—C4A—H4A	109.2	C5B—C6B—H64	110.3
C3A—C4A—C5A	110.49 (12)	H63—C6B—H64	108.5
C3A—C4A—H4A	109.2	O6B—C7B—C8B	104.16 (11)
C5A—C4A—H4A	109.2	O6B—C7B—C14B	110.02 (11)
O1A—C5A—C4A	109.63 (12)	O6B—C7B—C20B	108.11 (11)
O1A—C5A—C6A	107.11 (12)	C8B—C7B—C14B	105.82 (11)
O1A—C5A—H5A	109.8	C20B—C7B—C8B	112.91 (11)
C6A—C5A—C4A	110.63 (12)	C20B—C7B—C14B	115.25 (12)
C6A—C5A—H5A	109.8	C9B—C8B—C7B	120.04 (14)
C4A—C5A—H5A	109.8	C13B—C8B—C7B	121.55 (15)
O6A—C6A—C5A	108.56 (12)	C13B—C8B—C9B	118.26 (15)
O6A—C6A—H61	110.0	C8B—C9B—H9B	119.6
O6A—C6A—H62	110.0	C10B—C9B—C8B	120.88 (18)
C5A—C6A—H61	110.0	C10B—C9B—H9B	119.6
C5A—C6A—H62	110.0	C9B—C10B—H10B	119.7
H61—C6A—H62	108.4	C11B—C10B—C9B	120.7 (2)
O6A—C7A—C8A	107.53 (11)	C11B—C10B—H10B	119.7
O6A—C7A—C14A	105.38 (11)	C10B—C11B—C12B	119.58 (17)
O6A—C7A—C20A	110.27 (11)	C10B—C11B—H11B	120.2
C8A—C7A—C20A	115.29 (12)	C12B—C11B—H11B	120.2
C14A—C7A—C8A	112.45 (11)	C11B—C12B—C13B	120.3 (2)
C14A—C7A—C20A	105.50 (11)	C11B—C12B—H12B	119.8
C9A—C8A—C7A	122.39 (14)	C13B—C12B—H12B	119.8
C9A—C8A—C13A	117.77 (15)	C8B—C13B—C12B	120.29 (19)
C13A—C8A—C7A	119.04 (13)	C8B—C13B—H13B	119.9
C8A—C9A—H9A	119.5	C12B—C13B—H13B	119.9
C10A—C9A—C8A	120.97 (17)	C15B—C14B—C7B	120.41 (13)
C10A—C9A—H9A	119.5	C15B—C14B—C19B	118.08 (14)
C9A—C10A—H10A	119.7	C19B—C14B—C7B	121.26 (13)
C11A—C10A—C9A	120.59 (19)	C14B—C15B—H15B	119.7
C11A—C10A—H10A	119.7	C16B—C15B—C14B	120.60 (16)
C10A—C11A—C12A	119.30 (18)	C16B—C15B—H15B	119.7
C10A—C11A—H11A	120.3	C15B—C16B—H16B	119.6
C12A—C11A—H11A	120.3	C17B—C16B—C15B	120.80 (17)
C11A—C12A—C13A	120.31 (19)	C17B—C16B—H16B	119.6
C11A—C12A—H12A	119.8	C16B—C17B—C18B	119.20 (16)
C13A—C12A—H12A	119.8	C16B—C17B—H17B	120.4
C8A—C13A—H13A	119.5	C18B—C17B—H17B	120.4
C12A—C13A—C8A	121.05 (17)	C17B—C18B—C19B	120.24 (17)
C12A—C13A—H13A	119.5	C17B—C18B—H18B	119.9
C15A—C14A—C7A	120.58 (14)	C19B—C18B—H18B	119.9
C19A—C14A—C7A	120.79 (14)	C14B—C19B—H19B	119.5
C19A—C14A—C15A	118.41 (15)	C18B—C19B—C14B	121.07 (16)
C14A—C15A—H15A	119.8	C18B—C19B—H19B	119.5
C16A—C15A—C14A	120.39 (17)	C21B—C20B—C7B	123.23 (14)
C16A—C15A—H15A	119.8	C21B—C20B—C25B	117.62 (15)
C15A—C16A—H16A	119.5	C25B—C20B—C7B	118.53 (14)
C17A—C16A—C15A	120.95 (18)	C20B—C21B—C22B	120.69 (18)
C17A—C16A—H16A	119.5	C20B—C21B—H21B	119.7
C16A—C17A—H17A	120.5	C22B—C21B—H21B	119.7
C18A—C17A—C16A	119.01 (17)	C21B—C22B—H22B	119.6
C18A—C17A—H17A	120.5	C23B—C22B—C21B	120.79 (19)
C17A—C18A—C19A	120.73 (18)	C23B—C22B—H22B	119.6
C17A—C18A—H18A	119.6	C24B—C23B—C22B	119.11 (17)
C19A—C18A—H18A	119.6	C24B—C23B—H23B	120.4
C14A—C19A—C18A	120.49 (17)	C22B—C23B—H23B	120.4
C14A—C19A—H19A	119.8	C23B—C24B—C25B	120.55 (19)
C18A—C19A—H19A	119.8	C23B—C24B—H24B	119.7
C21A—C20A—C7A	121.38 (14)	C25B—C24B—H24B	119.7
C25A—C20A—C7A	119.85 (14)	C20B—C25B—H25B	119.4
C25A—C20A—C21A	118.44 (15)	C24B—C25B—C20B	121.24 (18)
C20A—C21A—H21A	120.0	C24B—C25B—H25B	119.4
C22A—C21A—C20A	119.97 (17)	O2B—C26B—H264	109.5
C22A—C21A—H21A	120.0	O2B—C26B—H265	109.5
C21A—C22A—H22A	119.5	O2B—C26B—H266	109.5
C23A—C22A—C21A	121.01 (19)	H264—C26B—H265	109.5
C23A—C22A—H22A	119.5	H264—C26B—H266	109.5
C22A—C23A—C24A	119.63 (17)	H265—C26B—H266	109.5
C22A—C23A—H23A	120.2	C27—O7—H71	107.5 (18)
C24A—C23A—H23A	120.2	C28—O8—H81	106.8 (17)
C23A—C24A—C25A	120.72 (19)	C29—O9—H91	107 (3)
C23A—C24A—H24A	119.6	C30—O10—H101	111 (2)
C25A—C24A—H24A	119.6	O7—C27—H271	109.5
C20A—C25A—H25A	119.9	O7—C27—H272	109.5
C24A—C25A—C20A	120.18 (17)	O7—C27—H273	109.5
C24A—C25A—H25A	119.9	H271—C27—H272	109.5
O2A—C26A—H261	109.5	H271—C27—H273	109.5
O2A—C26A—H263	109.5	H272—C27—H273	109.5
O2A—C26A—H262	109.5	O8—C28—H281	109.5
H261—C26A—H262	109.5	O8—C28—H282	109.5
H261—C26A—H263	109.5	O8—C28—H283	109.5
H263—C26A—H262	109.5	H281—C28—H282	109.5
C1B—O1B—C5B	114.55 (11)	H281—C28—H283	109.5
C1B—O2B—C26B	112.38 (14)	H282—C28—H283	109.5
C2B—O3B—H32	106.1 (16)	O9—C29—H291	109.5
C3B—O4B—H42	104.3 (17)	O9—C29—H292	109.5
C4B—O5B—H52	110.0 (19)	O9—C29—H293	109.5
C6B—O6B—C7B	117.24 (10)	H291—C29—H292	109.5
O1B—C1B—C2B	110.24 (12)	H291—C29—H293	109.5
O1B—C1B—H1B	108.4	H292—C29—H293	109.5
O2B—C1B—O1B	111.98 (12)	O10—C30—H301	109.5
O2B—C1B—C2B	109.19 (13)	O10—C30—H302	109.5
O2B—C1B—H1B	108.4	O10—C30—H303	109.5
C2B—C1B—H1B	108.4	H301—C30—H302	109.5
O3B—C2B—C1B	108.99 (13)	H301—C30—H303	109.5
O3B—C2B—C3B	112.72 (13)	H302—C30—H303	109.5
			
C5A—O1A—C1A—O2A	60.07 (16)	C5B—O1B—C1B—O2B	62.92 (16)
C5A—O1A—C1A—C2A	−61.44 (16)	C5B—O1B—C1B—C2B	−58.85 (16)
C1A—O1A—C5A—C4A	61.81 (16)	C1B—O1B—C5B—C4B	59.91 (15)
C1A—O1A—C5A—C6A	−178.11 (13)	C1B—O1B—C5B—C6B	−177.62 (12)
C7A—O6A—C6A—C5A	−167.89 (12)	C7B—O6B—C6B—C5B	168.28 (11)
C6A—O6A—C7A—C8A	−53.73 (15)	C6B—O6B—C7B—C8B	167.83 (11)
C6A—O6A—C7A—C14A	−173.89 (11)	C6B—O6B—C7B—C14B	−79.14 (14)
C6A—O6A—C7A—C20A	72.73 (15)	C6B—O6B—C7B—C20B	47.50 (15)
O1A—C1A—O2A—C26A	62.37 (17)	O1B—C1B—O2B—C26B	65.80 (17)
C2A—C1A—O2A—C26A	−175.89 (14)	C2B—C1B—O2B—C26B	−171.82 (14)
O1A—C1A—C2A—O3A	177.58 (12)	O2B—C1B—C2B—O3B	55.92 (16)
O1A—C1A—C2A—C3A	54.39 (17)	O1B—C1B—C2B—O3B	179.34 (12)
O2A—C1A—C2A—O3A	54.24 (16)	O2B—C1B—C2B—C3B	−69.41 (15)
O2A—C1A—C2A—C3A	−68.95 (16)	O1B—C1B—C2B—C3B	54.00 (16)
O3A—C2A—C3A—O4A	66.55 (16)	O3B—C2B—C3B—O4B	64.53 (16)
O3A—C2A—C3A—C4A	−172.44 (13)	O3B—C2B—C3B—C4B	−174.97 (13)
C1A—C2A—C3A—O4A	−171.50 (13)	C1B—C2B—C3B—O4B	−172.22 (12)
C1A—C2A—C3A—C4A	−50.49 (17)	C1B—C2B—C3B—C4B	−51.71 (16)
O4A—C3A—C4A—O5A	−71.51 (16)	O4B—C3B—C4B—O5B	−70.07 (15)
O4A—C3A—C4A—C5A	168.67 (12)	O4B—C3B—C4B—C5B	170.22 (12)
C2A—C3A—C4A—O5A	170.16 (13)	C2B—C3B—C4B—O5B	171.88 (12)
C2A—C3A—C4A—C5A	50.34 (17)	C2B—C3B—C4B—C5B	52.17 (15)
O5A—C4A—C5A—O1A	−174.91 (12)	O5B—C4B—C5B—O1B	−175.70 (11)
O5A—C4A—C5A—C6A	67.18 (16)	O5B—C4B—C5B—C6B	65.41 (15)
C3A—C4A—C5A—O1A	−54.15 (16)	C3B—C4B—C5B—O1B	−55.10 (14)
C3A—C4A—C5A—C6A	−172.05 (13)	C3B—C4B—C5B—C6B	−173.99 (12)
O1A—C5A—C6A—O6A	68.42 (15)	O1B—C5B—C6B—O6B	−70.33 (14)
C4A—C5A—C6A—O6A	−172.14 (12)	C4B—C5B—C6B—O6B	50.52 (16)
O6A—C7A—C8A—C9A	96.59 (16)	O6B—C7B—C8B—C9B	27.35 (18)
O6A—C7A—C8A—C13A	−72.94 (16)	O6B—C7B—C8B—C13B	−157.16 (14)
C14A—C7A—C8A—C9A	−147.85 (14)	C14B—C7B—C8B—C9B	−88.66 (17)
C14A—C7A—C8A—C13A	42.63 (18)	C14B—C7B—C8B—C13B	86.84 (17)
C20A—C7A—C8A—C9A	−26.9 (2)	C20B—C7B—C8B—C9B	144.39 (14)
C20A—C7A—C8A—C13A	163.62 (13)	C20B—C7B—C8B—C13B	−40.12 (19)
O6A—C7A—C14A—C15A	−23.67 (18)	O6B—C7B—C14B—C15B	−10.25 (17)
O6A—C7A—C14A—C19A	161.74 (14)	O6B—C7B—C14B—C19B	175.57 (13)
C8A—C7A—C14A—C15A	−140.52 (15)	C8B—C7B—C14B—C15B	101.71 (15)
C8A—C7A—C14A—C19A	44.89 (19)	C8B—C7B—C14B—C19B	−72.47 (16)
C20A—C7A—C14A—C15A	93.02 (16)	C20B—C7B—C14B—C15B	−132.76 (14)
C20A—C7A—C14A—C19A	−81.57 (17)	C20B—C7B—C14B—C19B	53.06 (18)
O6A—C7A—C20A—C21A	−168.10 (13)	O6B—C7B—C20B—C21B	−103.70 (17)
O6A—C7A—C20A—C25A	18.56 (17)	O6B—C7B—C20B—C25B	67.09 (16)
C8A—C7A—C20A—C21A	−46.12 (18)	C8B—C7B—C20B—C21B	141.61 (16)
C8A—C7A—C20A—C25A	140.54 (14)	C8B—C7B—C20B—C25B	−47.60 (18)
C14A—C7A—C20A—C21A	78.59 (16)	C14B—C7B—C20B—C21B	19.8 (2)
C14A—C7A—C20A—C25A	−94.76 (15)	C14B—C7B—C20B—C25B	−169.38 (13)
C7A—C8A—C9A—C10A	−168.77 (15)	C7B—C8B—C9B—C10B	175.70 (15)
C13A—C8A—C9A—C10A	0.9 (2)	C13B—C8B—C9B—C10B	0.1 (3)
C7A—C8A—C13A—C12A	169.03 (15)	C7B—C8B—C13B—C12B	−174.68 (16)
C9A—C8A—C13A—C12A	−1.0 (2)	C9B—C8B—C13B—C12B	0.9 (3)
C8A—C9A—C10A—C11A	0.0 (3)	C8B—C9B—C10B—C11B	−1.0 (3)
C9A—C10A—C11A—C12A	−0.7 (3)	C9B—C10B—C11B—C12B	1.0 (3)
C10A—C11A—C12A—C13A	0.6 (3)	C10B—C11B—C12B—C13B	0.0 (3)
C11A—C12A—C13A—C8A	0.2 (3)	C11B—C12B—C13B—C8B	−0.9 (3)
C7A—C14A—C15A—C16A	−174.47 (15)	C7B—C14B—C15B—C16B	−175.31 (14)
C19A—C14A—C15A—C16A	0.2 (3)	C19B—C14B—C15B—C16B	−1.0 (2)
C7A—C14A—C19A—C18A	173.60 (17)	C7B—C14B—C19B—C18B	174.75 (15)
C15A—C14A—C19A—C18A	−1.1 (3)	C15B—C14B—C19B—C18B	0.4 (2)
C14A—C15A—C16A—C17A	0.9 (3)	C14B—C15B—C16B—C17B	0.6 (3)
C15A—C16A—C17A—C18A	−1.3 (3)	C15B—C16B—C17B—C18B	0.2 (3)
C16A—C17A—C18A—C19A	0.4 (3)	C16B—C17B—C18B—C19B	−0.7 (3)
C17A—C18A—C19A—C14A	0.8 (3)	C17B—C18B—C19B—C14B	0.4 (3)
C7A—C20A—C21A—C22A	−174.82 (15)	C7B—C20B—C21B—C22B	170.08 (16)
C25A—C20A—C21A—C22A	−1.4 (2)	C25B—C20B—C21B—C22B	−0.8 (3)
C7A—C20A—C25A—C24A	175.73 (15)	C7B—C20B—C25B—C24B	−170.56 (17)
C21A—C20A—C25A—C24A	2.2 (2)	C21B—C20B—C25B—C24B	0.8 (3)
C21A—C22A—C23A—C24A	1.6 (3)	C20B—C21B—C22B—C23B	0.4 (3)
C20A—C21A—C22A—C23A	−0.5 (3)	C21B—C22B—C23B—C24B	0.1 (3)
C22A—C23A—C24A—C25A	−0.8 (3)	C22B—C23B—C24B—C25B	−0.1 (3)
C23A—C24A—C25A—C20A	−1.1 (3)	C23B—C24B—C25B—C20B	−0.3 (3)

### Hydrogen-bond geometry (Å, º)

**Table:**

*D*—H···*A*	*D*—H	H···*A*	*D*···*A*	*D*—H···*A*
O3*A*—H31···O10	0.84 (3)	1.88 (3)	2.707 (2)	169 (2)
O3*B*—H32···O7	0.82 (3)	1.91 (3)	2.713 (2)	165 (3)
O4*A*—H41···O2*B*	0.83 (3)	2.02 (2)	2.8287 (18)	164.5 (19)
O4*B*—H42···O2*A*^i^	0.77 (3)	2.15 (3)	2.9046 (19)	166 (3)
O5*A*—H51···O8	0.79 (3)	1.87 (3)	2.667 (2)	175 (4)
O5*B*—H52···O9^i^	0.81 (4)	1.90 (4)	2.704 (2)	173 (3)
O7—H71···O5*A*^i^	0.79 (2)	1.98 (2)	2.763 (2)	170 (3)
O8—H81···O3*B*	0.90 (3)	1.84 (3)	2.727 (2)	170 (3)
O9—H91···O3*A*	0.85 (4)	1.91 (4)	2.741 (2)	167 (4)
O10—H101···O5*B*	0.81 (3)	1.95 (3)	2.754 (2)	170 (3)
C1*B*—H1*B*···O3*A*^ii^	0.98	2.32	3.2469 (19)	157
C3*A*—H3*A*···O4*B*^iii^	0.98	2.59	3.471 (2)	149
C3*B*—H3*B*···O4*A*	0.98	2.49	3.391 (2)	153
C15*B*—H15*B*···O1*B*	0.93	2.52	3.417 (2)	161
C25*A*—H25*A*···O1*A*	0.93	2.60	3.443 (2)	151

Symmetry codes: (i) *x*−1, *y*, *z*; (ii) *x*, *y*−1, *z*; (iii) *x*+1, *y*, *z*.
